# Evaluating the Accuracy, Readability, and Consistency of Artificial Intelligence Models in Patient Education for Rheumatoid Arthritis, Osteoarthritis, and Psoriatic Arthritis

**DOI:** 10.7759/cureus.106160

**Published:** 2026-03-30

**Authors:** Morgan Lilly, Kanika Monga

**Affiliations:** 1 Department of Engineering Medicine, Texas A&amp;M University, Houston, USA; 2 Department of Rheumatology, Houston Methodist Academic Medicine Associates - Rheumatology, Houston Methodist Hospital, Houston, USA

**Keywords:** artificial intelligence in healthcare, degenerative joint disease, information literacy, information quality, large language models, patient education material, psoriatic arthritis, rheumatoid arthritis

## Abstract

Introduction and aim: Artificial intelligence (AI) chatbots are increasingly used by patients to obtain medical information before seeking clinical care; however, the accuracy, readability, and consistency of AI-generated information in rheumatology remain uncertain. This study aimed to assess the readability, accuracy, and consistency of large language models (LLMs) generated patient education content for rheumatoid arthritis (RA), osteoarthritis (OA), and psoriatic arthritis (PsA).

Methods: From August 18, 2025, to August 24, 2025, three standardized patient-facing questions per disease were submitted daily to three LLMs, specifically ChatGPT (San Francisco, CA: OpenAI), Google Gemini (Mountain View, CA: Google LLC), and OpenEvidence (Cambridge, MA: OpenEvidence Inc.), with histories cleared between submissions. Readability (Hemingway grade level), word count, accuracy (on a 1-5 scale), and day-to-day consistency (Jaccard similarity) were measured. Responses from each model’s most consistent day were accuracy-rated by rheumatology fellows and attendings.

Results: A total of 189 responses were collected. No model consistently met the American Medical Association (AMA)/National Institutes of Health (NIH)-recommended reading levels for sixth through eighth grade. OpenEvidence produced the most technical content (≥17th grade, indicating postbaccalaureate readability), while ChatGPT and Gemini averaged 11.9-12.0 grade. Gemini generated the longest responses (>500 words). ChatGPT showed the highest day-to-day stability (range: <0.07), Gemini moderate variability, and OpenEvidence both the widest range (0.24) and the highest average similarity (0.383). Accuracy ratings varied as follows: OpenEvidence generally scored higher for RA and PsA, while ChatGPT and Gemini were similar across diseases. OA responses showed minimal difference. Best and worst responses mirrored these trends.

Conclusions: Current LLMs generate rheumatology information above the recommended reading levels. ChatGPT was the most consistent, Gemini the most detailed, and OpenEvidence the most technical. Persistent barriers to readability highlight the need for health-literacy-optimized AI communication.

## Introduction

Artificial intelligence (AI)-powered large language models (LLMs) are increasingly consulted by patients seeking health information before professional evaluation. As AI tools become embedded in daily life, from decision-making about finances and education to troubleshooting household problems, patients are now extending this reliance to medical diagnosis, symptom interpretation, and disease management. While the use of these tools in society can improve access to information, significant concerns remain regarding the accuracy, readability, and consistency of AI-generated responses, particularly when applied to complex medical domains. In this context, it is notable that major organizations such as the American Medical Association and the National Institutes of Health recommend that patient education materials be written at a sixth through eighth-grade reading level, highlighting the importance of appropriately tailored content for the general public [1,2].

This issue is especially relevant in rheumatology, as rheumatologic diseases are multifaceted and often difficult for patients to conceptualize, as they involve immunologic, genetic, biomechanical, and environmental components. Rheumatologic conditions such as rheumatoid arthritis (RA), osteoarthritis (OA), and psoriatic arthritis (PsA) are increasingly recognized as complex, systemic diseases rather than simple mechanical or uniform immune disorders. For example, Gravallese and Firestein described RA as a disease with common origins but divergent mechanisms, involving multiple immunologic, genetic, and environmental pathways [3]. Similarly, the paradigm of OA is shifting as follows: what was long regarded as "wear-and-tear" is now understood as a systemic disease implicating metabolic dysfunction, extracellular-matrix degradation, and innate immune activation [4]. Finally, PsA exemplifies multifactorial pathogenesis as follows: genetic susceptibility, environmental influences, and complex immune dysregulation (both innate and adaptive) often contribute to inflammation, bone and cartilage damage, and heterogeneous clinical phenotypes [5]. These complexities pose substantial challenges for patient understanding, particularly for individuals with limited health literacy, and underscore the importance of clear, accurate educational resources.

Prior research has demonstrated both the promise and limitations of AI in medicine. Kuroiwa et al. evaluated ChatGPT (San Francisco, CA: OpenAI) as a self-diagnostic tool in orthopedic conditions, noting variability in reliability [6]. Kara et al. compared ChatGPT, Gemini, and Perplexity (San Francisco, CA: Perplexity AI, Inc.) on ankylosing spondylitis, highlighting platform-dependent differences in readability and quality [7]. Uz and Umay questioned the reliability of ChatGPT for rheumatology-focused information, while other studies explored the use of AI in education on osteoarthritis and psoriatic disease [8].

Despite these efforts, little systematic work has compared multiple AI models for addressing patient-facing questions about rheumatoid arthritis (RA), osteoarthritis (OA), and psoriatic arthritis (PsA), conditions in which health literacy gaps are common and educational clarity is essential. This study evaluates ChatGPT, Google Gemini (Mountain View, CA: Google LLC), and OpenEvidence (Cambridge, MA: OpenEvidence Inc.) for readability, word count, accuracy, day-to-day consistency, and qualitative characteristics of patient-facing responses.

## Materials and methods

Study design and timeline

This observational evaluation was conducted over a fixed one-week period from August 18, 2025, to August 24, 2025. Given that no human subjects or patient data were involved, this study did not require institutional review board (IRB) oversight. Three standardized patient-facing questions targeting common informational needs in rheumatology were developed for each of the following three disease categories: RA, OA, and PsA. Each question was submitted independently and sequentially to the following three LLMs: ChatGPT, Google Gemini, and OpenEvidence. To minimize the potential for contextual carryover or learning effects between queries, all conversation histories and contextual memory were cleared immediately prior to each submission. Data collection was performed at approximately the same time each day to account for potential diurnal variability in model generation.

Data collection

The three standardized questions were selected for their face validity in representing core informational domains relevant to patient education, specifically focusing on disease definition, treatment options, and prognosis. The exact questions asked were “what is this disease?,” “how is it treated?,” and “what is the prognosis?”. These questions were designed to be generalizable and representative of common inquiries from patients or members of the public. Each model’s response to each question was recorded in full text and stored in a secure database for future reference and subsequent analysis.

Metrics and analysis

All responses were evaluated using predefined quantitative and qualitative metrics. Word count was calculated for each response to assess verbosity and potential information density. Readability was assessed using the Hemingway grade level, an established measure of textual complexity, and compared with the American Medical Association and National Institutes of Health recommendations for patient-facing health communication at the sixth- to eighth-grade reading level.

To evaluate accuracy, the day with the highest internal textual similarity, assessed by Jaccard score, was identified for each model. Responses from that day were independently rated by eight clinicians (rheumatology fellows and attendings) using a five-point accuracy scale based on clinical knowledge as follows: (1) completely inaccurate - factually wrong, misleading, or potentially harmful if applied clinically, (2) mostly inaccurate - major errors or omissions; overall unreliable, (3) partially accurate - some correct information but incomplete or partially incorrect; needs major revision, (4) mostly accurate - generally, correct with only minor errors or omissions, (5) completely accurate - factually correct, complete, and clinically appropriate. Individual reviewer scores were averaged to generate a final accuracy score for each response. This approach allowed for independent assessment while reducing subjectivity through aggregation (tables in appendix).

Day-to-day consistency was assessed using Jaccard similarity scores, which quantify how much the content of responses overlaps from one day to the next by comparing each word. Scores range from 0 (no overlap) to 1.0 (complete overlap), allowing the evaluation of how stable each model’s responses were over time.

Statistical analysis and qualitative evaluation

Descriptive statistics were calculated for all quantitative measures. One-way analysis of variance (ANOVA) was used to test for differences in readability and word count across the three LLMs. Accuracy scores were compared descriptively based on observed differences in mean ratings across models. In addition to quantitative assessment, responses were reviewed qualitatively for elements of patient-facing clarity, including the use of formatting features (e.g., headings, bullet points), the presence of explicit examples or analogies, and the citation of sources or references. This mixed-methods approach allowed both numerical comparisons and contextual interpretations of educational value.

Response rate

Based on the study design (three LLMs × three questions × seven days), 189 responses were expected. All 189 responses were successfully collected, yielding a 100% response rate. No responses were missing or excluded from the analysis. This complete dataset ensured consistency across models and time points, allowing for a standardized comparison of outputs.

## Results

Readability

Readability varied substantially across LLM platforms, and none met the AMA/NIH-recommended sixth- to eighth-grade levels for patient-facing materials (Tables 1-3). OpenEvidence responses were consistent at the postbaccalaureate level (≥ 17th-grade readability), reflecting highly technical language and complex sentence structures. ChatGPT responses ranged from eighth grade to postgraduate level, with a mean grade level of 11.94, while Gemini responses ranged from ninth to 14th grade, with a mean grade level of 11.93. The slightly narrower range of responses seen with Gemini suggests greater consistency in language and sentence structure across responses than with ChatGPT. Analysis of variance (ANOVA) confirmed significant differences in readability across platforms (p<0.05). When OpenEvidence was excluded, ChatGPT and Gemini were largely comparable, suggesting that their responses were more similar in terms of readability.

**Table 1:** Readability, response length, and consistency results for ChatGPT responses. Q# refers to the corresponding question number. Scores ≥17 represent postbaccalaureate reading level. ChatGPT (San Francisco, CA: OpenAI)

Variables	Rheumatoid arthritis			Osteoarthritis			Psoriatic arthritis		
	Q1	Q2	Q3	Q1	Q2	Q3	Q1	Q2	Q3
Average readability (Hemingway)	10.43	13.86	11.86	10.00	13.57	11.57	10.71	13.00	12.43
High readability level (Hemingway)	12.00	≥17	13.00	13.00	≥17	≥17	12.00	15.00	14.00
Low readability level (Hemingway)	9.00	12.00	11.00	8.00	11.00	10.00	9.00	9.00	11.00
Average response length (word count)	244.71	344.86	313.86	282.86	337.43	310.71	303.71	376.43	295.00
Consistency across days (Jaccard similarity)	0.28	0.26	0.28	0.24	0.26	0.31	0.29	0.29	0.29

**Table 2:** Readability, response length, and consistency results for Google Gemini responses. Q# refers to the corresponding question number. Google Gemini (Mountain View, CA: Google LLC)

Variables	Rheumatoid arthritis			Osteoarthritis			Psoriatic arthritis		
	Q1	Q2	Q3	Q1	Q2	Q3	Q1	Q2	Q3
Average readability (Hemingway)	10.43	12.43	13.00	9.71	11.86	11.57	12.00	13.29	13.00
High readability level (Hemingway)	11.00	13.00	14.00	11.00	13.00	13.00	14.00	14.00	14.00
Low readability level (Hemingway)	10.00	12.00	12.00	9.00	11.00	10.00	11.00	12.00	11.00
Average response length (word count)	361.71	559.86	523.57	532.00	546.29	509.71	505.57	519.57	517.71
Consistency across days (Jaccard similarity)	0.44	0.40	0.32	0.41	0.40	0.29	0.42	0.39	0.32

**Table 3:** Readability, response length, and consistency results for OpenEvidence responses. Q# refers to the corresponding question number. Scores ≥17 represent postbaccalaureate reading level. OpenEvidence (Cambridge, MA: OpenEvidence Inc.)

Variables	Rheumatoid arthritis			Osteoarthritis			Psoriatic arthritis		
	Q1	Q2	Q3	Q1	Q2	Q3	Q1	Q2	Q3
Average readability (Hemingway)	≥17	≥17	≥17	≥17	≥17	≥17	≥17	≥17	≥17
High readability level (Hemingway)	≥17	≥17	≥17	≥17	≥17	≥17	≥17	≥17	≥17
Low readability level (Hemingway)	≥17	≥17	≥17	≥17	≥17	≥17	≥17	≥17	≥17
Average response length (word count)	266.43	478.00	259.57	226.86	294.14	261.14	251.00	315.43	259.71
Consistency across days (Jaccard similarity)	0.40	0.44	0.36	0.38	0.44	0.22	0.45	0.46	0.30

Word count

Response length differed significantly among models. Gemini consistently produced the longest responses, often exceeding 500 words, while OpenEvidence generated shorter but highly technical responses. ChatGPT yielded intermediate-length responses, balancing details and conciseness. ANOVA demonstrated significant differences in word count across platforms (p<0.05), reflecting distinct design priorities as follows: Gemini emphasizes comprehensive coverage, OpenEvidence emphasizes technical precision, and ChatGPT emphasizes clarity and readability.

Consistency

OpenEvidence had the widest range of day-to-day response similarity (Jaccard similarity range: 0.24) but the highest mean similarity (0.383), indicating variable yet internally consistent content. Gemini showed intermediate variability (range: 0.16, mean: 0.378), while ChatGPT responses were the most stable (range: ≤0.7) but had the lowest mean similarity (0.277), reflecting conservative but less content-rich outputs. Across all LLMs, prognosis-related questions were most variable, whereas disease definition and treatment questions elicited more consistent responses.

Accuracy

OpenEvidence achieved the highest mean accuracy across RA, OA, and PsA. Rheumatology reviewers awarded RA responses more 5/5 accuracy scores (6) across all models compared with OA (4) and PsA (5). OpenEvidence in particular received the highest number of 5/5 ratings, with five responses rated as fully accurate, compared with 3/5 for OA and PsA. For RA, OpenEvidence generally received higher ratings than ChatGPT and Gemini, while OA and PsA ratings were more evenly distributed across models. Best- or worst-case selections mirrored these trends as follows: OpenEvidence was preferred for RA, whereas OA and PsA selections were more evenly distributed (Table 4). Gemini received the most “worst” response votes for RA and PsA, and ChatGPT for OA.

**Table 4:** Expert ratings of LLM responses (best/worst) by rheumatologic disease category. ChatGPT (San Francisco, CA: OpenAI), Google Gemini (Mountain View, CA: Google LLC), and OpenEvidence (Cambridge, MA: OpenEvidence Inc.) LLM: large language model

Variables	Question	ChatGPT	Gemini	OpenEvidence
Votes for best response	What is RA?	1	1	6
	What is OA?	2	3	3
	What is PsA?	2	3	3
Votes for worst response	What is RA?	3	4	1
	What is OA?	3	3	2
	What is PsA?	2	4	2

Qualitative observations

OpenEvidence responses were dense, heavily citation-based, and organized in long paragraphs. In contrast, ChatGPT and Gemini employed structured formats with headers, bullet points, and concise summaries, enhancing readability and facilitating rapid information extraction for users.

## Discussion

This study compared three widely used large language models, namely ChatGPT, Google Gemini, and OpenEvidence, in their ability to generate patient-facing rheumatology education material for rheumatoid arthritis, osteoarthritis, and psoriatic arthritis. Across all models, responses demonstrated meaningful differences in length, structure, technicality, and consistency. ChatGPT offered the most readable and structurally approachable responses, with moderate-length outputs, the lowest day-to-day variability, and occasional near-target readability (~8th grade). Features such as headers, bullet points, and summaries facilitate easier navigation and comprehension. ChatGPT also maintained the narrowest similarity range (<0.07), reflecting stable phrasing and predictable structure. OpenEvidence produced the most technically rigorous, citation-supported responses, characterized by dense biomedical phrasing and consistent postgraduate-level readability. This platform generally received the highest accuracy ratings across, but its day-to-day consistency showed the widest range (0.24), indicating individual outputs could vary substantially. Google Gemini produced the longest and most comprehensive responses, with frequent outputs exceeding 500 words and a moderate readability range (ninth to 14th grade). Its high word counts reflect its tendency to provide broad context and extensive elaboration, which may benefit users who prefer detailed explanations. However, this verbosity may overwhelm patients seeking concise guidance. Accuracy ratings were more evenly distributed across RA, OA, and PsA, though Gemini received the most “worst” response votes for RA and PsA. Gemini also displayed intermediate day-to-day consistency (range: 0.16, mean similarity: 0.378), indicating relatively stable but information-dense outputs.

Building on these observations, the results of this study highlight the tradeoffs between technical depth, detail, and accessibility. OpenEvidence, with its biomedical terminology and citation-style responses, achieved the highest accuracy ratings, consistent with its orientation toward expert-level content [5]. ChatGPT balanced readability and structural design with a moderate level of technicality, supporting efficient information-gathering and comprehension, consistent with previous research on AI-assisted patient communication [6]. Gemini produced highly detailed and context-rich explanations that may overwhelm patients with lower health literacy but align with prior findings showing that comprehensive AI outputs can increase perceived informational value [7]. These differences likely reflect a model’s underlying knowledge base, training objectives, and architecture, all of which influence how a system interacts with the audience. Therefore, variations in the resulting user interfaces or engagement suggest that different LLMs may target different audiences. For instance, findings from this study suggest that ChatGPT may be best suited for general patient education, OpenEvidence for clinicians, and Gemini for patients seeking detailed explanations.

Extending this comparison to the broader literature, these results align with trends reported in prior research on AI-generated medical information. Across multiple studies, LLMs have demonstrated the ability to generate high-quality explanations, achieving moderate to high accuracy when responding to medical questions [9,10]. Similarly, comparisons of AI-generated responses to physician answers suggest that LLMs can provide supplementary information and can therefore be useful for individuals seeking detailed information prior to or after a doctor’s appointment [11]. Furthermore, systematic reviews have highlighted the potential of LLMs, such as ChatGPT, to assist with patient education and information retrieval, while emphasizing the importance of ongoing evaluation of response accuracy and consistency in medical settings [12].

Another important observation from our study was variability in response consistency across repeated queries. ChatGPT demonstrated the narrowest day-to-day variation in phrasing and structure, whereas OpenEvidence showed a wider range of similarity scores, despite higher overall content overlap. This fluctuation aligns with prior research indicating that small differences in prompts or model updates can result in variations in AI-generated medical outputs [12]. For patients relying on AI as a primary source of information, such variability may result in confusion, underscoring the need for improved reliability in LLM models.

Despite differences in content structure, response length, and technical depth, a consistent finding across all models was elevated response readability. None of the systems reliably produced patient-facing explanations within the sixth- to eighth-grade reading level recommended by major health communication guidelines [1,2]. This finding is also consistent with prior work demonstrating that both the traditional online patient education materials and unprompted AI-generated medical content frequently exceed the recommended readability threshold [8]. Specifically, Uz and Umay analyzed ChatGPT-generated explanations for common rheumatic conditions and reported that, although some outputs were clear and structured, most responses still exceeded recommended reading levels [8].

At the same time, prior research indicates that LLMs can be leveraged to improve readability when explicitly prompted. For example, Will et al. demonstrated that LLM-assisted revisions of patient education materials can substantially reduce reading grade levels while preserving critical clinical information [13]. Similar improvements have been reported in orthopedic and vascular patient education content, where LLM-generated revisions lowered rhetoric complexity without substantially altering medical accuracy [14,15]. Because limited health literacy affects up to one-third of U.S. adults, overly complex educational materials may hinder comprehension, resulting in reduced engagement in the shared decision-making process and further exacerbating health disparities [16-18].

Future work

Future work should expand on this framework by incorporating a broader and more diverse set of patient-centered questions, evaluating additional AI models, and extending analyses over longer time periods. Assessing a wider range of questions would provide a more comprehensive understanding of LLM performance in real-world environments, while longitudinal analyses could capture variations in responses from model updates. Additionally, including non-English responses would allow for assessment of multilingual performance. This is important as language barriers can further impact information accessibility or health literacy. Finally, future work should explore strategies to optimize AI-generated content for patient comprehension. One potential approach could involve assessing patient reading comprehension via a short quiz saved to a patient’s profile and tailoring responses accordingly.

Limitations

This study has several limitations. First, only three LLMs were evaluated, and these results may not generalize to other platforms or align with future model updates. Second, the standardized questions used here capture common patient inquiries but do not capture the full variability or nuance of real-world patient information-seeking. Third, accuracy ratings were based on expert reviewers and therefore may include some degree of subjectivity, despite being derived from trained rheumatology clinicians. Fourth, analyses were conducted within a single week, and temporal drift in model behavior was not assessed. Lastly, responses were evaluated in English only, potentially limiting generalizability to non-English-speaking populations and not accounting for variability in translation across LLMs.

## Conclusions

ChatGPT, Google Gemini, and OpenEvidence all produced clinically relevant patient-facing content for rheumatoid arthritis, osteoarthritis, and psoriatic arthritis, but they differed in length, structure, technical depth, and consistency. ChatGPT provided the most readable and structurally organized responses with low day-to-day variability, making it well-suited for general patient education. OpenEvidence produced the most technically rigorous and citation-supported outputs with the highest expert-rated accuracy, but its dense language and variable day-to-day consistency suggest that it is better suited for clinicians. Google Gemini generated the longest responses, providing additional context that may benefit patients who seek detailed explanations, though its length and complexity may overwhelm readers with lower health literacy.

Despite these differences, a consistent limitation across all models was elevated readability levels, which exceeded the sixth- to eighth-grade recommendations set by major health communication guidelines. This finding aligns with prior work showing that traditional online patient education materials and AI-generated medical content frequently exceed recommended reading levels. Because limited health literacy affects a substantial portion of the adult population, complex materials may hinder comprehension, reduce engagement in shared decision-making, and increase health disparities. At the same time, emerging evidence suggests that large language models can improve readability when prompted, allowing revision of patient education content to reduce content complexity while preserving medical accuracy. Without targeted optimization for health literacy, LLM-generated content risks widening existing disparities in patient understanding and engagement. Future work should focus on model-level modifications that enable adjustable readability outputs or standardized responses aligned with AMA and NIH recommendations. One potential future approach may involve assessing patient reading comprehension via a short quiz saved to a patient’s profile and tailoring responses accordingly, which may further enhance educational effectiveness. Aligning AI-generated medical education with established health communication standards will be essential to realizing the potential of LLMs as equitable, patient-centered tools in rheumatology.

## Appendices

**Table 5:** Expert ratings of AI-generated rheumatology responses. Survey instructions - each item in the table is an AI-generated response to the question: “What is __?” (the blank is filled with rheumatoid arthritis, osteoarthritis, or psoriatic arthritis). Expert reviewers were asked to evaluate each response for accuracy, completeness, and clinical appropriateness. Ratings were assigned using the following five-point scale, indicating how correct, reliable, and clinically applicable the information is: (1) completely inaccurate - factually wrong, misleading, or potentially harmful if applied clinically. (2) mostly inaccurate - major errors or omissions; overall unreliable. (3) partially accurate - some correct information, but incomplete or partially incorrect; needs major revision. (4) mostly accurate - generally correct with only minor errors or omissions. (5) completely accurate - factually correct, complete, and clinically appropriate. NSAIDs: non-steroidal anti-inflammatory drugs

Question identifier	AI-generated response
Response 1 - What is RA?	Rheumatoid arthritis (RA) is a chronic autoimmune disease that primarily affects the joints. In RA, the body’s immune system mistakenly attacks the synovium, which is the lining of the membranes that surround your joints. This causes inflammation, pain, swelling, and stiffness. Over time, this inflammation can lead to joint damage, deformities, and loss of function. Key features: autoimmune nature - the immune system attacks the body’s own tissues. Symmetrical joint involvement - usually affects the same joints on both sides of the body (e.g., both wrists, both knees). Commonly affected joints - small joints in the hands and feet are often affected first, but larger joints can also be involved. Extra-articular manifestations - RA can affect other organs, causing fatigue, fever, anemia, lung problems, or heart issues. Chronic and progressive - without treatment, RA can lead to joint destruction and disability over time. Symptoms - persistent joint pain and swelling, morning stiffness lasting more than 30-60 min, warmth and redness over joints, fatigue, and general malaise. Causes and risk factors - exact cause is unknown, but genetic, environmental (like smoking), and hormonal factors contribute. Treatment: medications - disease-modifying antirheumatic drugs (DMARDs), biologics, NSAIDs, and corticosteroids. Lifestyle - exercise, physical therapy, and joint protection techniques. Surgery - sometimes needed for joint replacement if severe damage occurs. RA is different from osteoarthritis (OA), which is mainly due to wear-and-tear rather than an autoimmune process.
Response 2 - What is RA?	Rheumatoid arthritis (RA) is a chronic autoimmune disease that causes inflammation of the joints. Unlike osteoarthritis, which is caused by "wear and tear" on the joints, RA is a condition where the body's immune system mistakenly attacks the healthy tissue that lines the joints. RA most commonly affects the small joints of the hands, wrists, and feet, but it can also affect larger joints like the knees, elbows, shoulders, and ankles. The disease typically affects the same joints on both sides of the body. Symptoms may develop slowly over time and can include joint pain, swelling, and stiffness, often worse in the morning or after periods of inactivity. Morning stiffness from RA can last for more than an hour, which is a key difference from other types of arthritis. Other symptoms include joint tenderness, warmth, and redness; fatigue, fever, and loss of appetite; rheumatoid nodules (firm bumps under the skin near affected joints); and deformity of joints over time. Inflammation associated with RA can also affect other parts of the body, including the eyes, lungs, heart, and skin. The symptoms of RA can vary in severity and may come and go, with periods of increased symptoms called "flares" and periods of less or no swelling and pain called "remission." The exact cause of RA is unknown, but is considered a combination of genetic and environmental factors. Risk factors include certain genes, environmental triggers such as cigarette smoke or infections, and hormonal factors, as RA is more common in women. There is no cure, but early diagnosis and treatment can help manage symptoms, reduce joint damage, and improve quality of life.
Response 3 - What is RA?	Rheumatoid arthritis is a chronic, systemic autoimmune disease characterized by persistent inflammation of the synovial joints, leading to pain, swelling, stiffness, and progressive joint destruction. The disease most commonly affects the small joints of the hands and feet in a symmetrical pattern, but any synovial joint can be involved as the disease progresses. Extra-articular manifestations are common and may include involvement of the skin, eyes, lungs, cardiovascular system, and other organs. Pathophysiologically, rheumatoid arthritis is driven by immune-mediated synovial inflammation, with infiltration of T cells, B cells, and monocytes, and the production of autoantibodies such as rheumatoid factor (RF) and anti-cyclic citrullinated peptide antibodies (ACPAs). These processes result in synovial hyperplasia (pannus formation), cartilage degradation, and bone erosion, mediated by pro-inflammatory cytokines, including tumor necrosis factor (TNF) and interleukin-6 (IL-6). The etiology is multifactorial, involving genetic predisposition (notably HLA-DRB1 alleles) and environmental factors such as smoking and certain infections. The prevalence is approximately 0.5-1% worldwide, with a female predominance and peak incidence in the third to fifth decades of life. Clinically, patients present with joint pain, morning stiffness lasting ≥30 min, and swelling, often accompanied by constitutional symptoms. Diagnosis is based on clinical assessment, serologic markers (RF, ACPA), and imaging. The American College of Rheumatology and the European Alliance of Associations for Rheumatology recommend a treat-to-target approach, aiming for remission or low disease activity using disease-modifying antirheumatic drugs (DMARDs).
Response 1 - What is OA?	Osteoarthritis (OA) is the most common form of arthritis, often described as a degenerative joint disease. It occurs when the protective cartilage that cushions the ends of your bones gradually wears down over time. This leads to pain, stiffness, swelling, and reduced movement in the affected joints. OA usually develops slowly and worsens with age, although it can also result from joint injury or overuse. Causes/risk factors: aging - natural wear and tear of cartilage. Joint injury or overuse - sports injuries, repetitive movements, or heavy physical work. Obesity - extra weight puts more stress on weight-bearing joints like knees and hips. Genetics - family history can increase risk. Other conditions - certain metabolic disorders or inflammatory conditions. Commonly affected joints - knees, hips, hands (especially the thumb and finger joints), spine (lower back and neck). Symptoms - joint pain that worsens with activity and improves with rest, stiffness especially in the morning or after inactivity, swelling or tenderness around the joint, reduced flexibility, or range of motion, sometimes a grating sensation or bone spurs. Diagnosis - physical exam, imaging - X-rays or MRI to look for cartilage loss, bone spurs, or joint space narrowing, sometimes lab tests to rule out other types of arthritis. Treatment: lifestyle - exercise, weight management, physical therapy. Medications - pain relievers (acetaminophen), NSAIDs, topical creams. Assistive devices - braces, shoe inserts, or walking aids. Procedures - corticosteroid injections, hyaluronic acid injections. Surgery - joint replacement in severe cases. OA is different from rheumatoid arthritis (RA), which is an autoimmune disease; OA is primarily mechanical and degenerative rather than inflammatory.
Response 2 - What is OA?	Osteoarthritis (OA) is the most common form of arthritis, affecting millions of people worldwide. It is a degenerative joint disease that occurs when the protective cartilage that cushions the ends of your bones wears down over time. This "wear and tear" on the joints leads to pain, stiffness, and loss of function. How osteoarthritis develops: in a healthy joint, cartilage is a smooth, slippery tissue that allows bones to move against each other with very little friction. With osteoarthritis, the cartilage becomes rough and breaks down. Eventually, if the cartilage wears away completely, the bones can rub directly against each other. This process also causes changes in the bone, such as the formation of bone spurs, and can lead to inflammation of the joint lining. Symptoms of osteoarthritis: OA symptoms typically develop slowly over many years and can vary in severity. The condition most commonly affects the joints that bear weight, such as the knees, hips, and spine, but can also affect the hands, neck, and feet. Common symptoms include pain: aching or throbbing pain in the affected joint that may worsen during or after movement; stiffness: joint stiffness most noticeable in the morning or after a period of inactivity, usually lasting less than 30 min; tenderness: the joint may feel tender when light pressure is applied; loss of flexibility: inability to move the joint through its full range of motion; grating sensation: a grating or popping sensation when using the joint; bone spurs: hard lumps may form around the affected joint. Causes and risk factors: while aging is a primary risk factor, osteoarthritis is not inevitable. Risk factors include age: risk increases with age; obesity: extra weight stresses weight-bearing joints; joint injury or overuse: prior injury or repetitive stress; genetics: inherited tendency; gender: women are more likely to develop OA. Diagnosis and treatment: OA is diagnosed based on physical exam, symptom review, and imaging (X-rays). Treatments help manage pain and improve joint function and may include medications (acetaminophen, NSAIDs, or prescription options), physical and occupational therapy (strengthening muscles, improving range of motion, teaching daily activity strategies), lifestyle changes (maintaining healthy weight, low-impact exercise), supportive devices (braces, shoe inserts, canes), and surgery for severe cases where other treatments fail.
Response 3 - What is OA?	Osteoarthritis is a chronic, progressive joint disease characterized by the degeneration of articular cartilage, subchondral bone remodeling, osteophyte formation, and variable degrees of synovial inflammation. It is a whole-joint disorder, affecting not only cartilage but also bone, synovium, ligaments, meniscus, and periarticular tissues. The pathogenesis involves a complex interplay of mechanical, metabolic, genetic, and inflammatory factors, with risk factors including older age, female sex, obesity, prior joint injury, and joint malalignment. Clinically, osteoarthritis presents with joint pain that is typically worsened by activity and relieved by rest, morning stiffness lasting less than 30 min, and functional limitation. Physical findings may include bony enlargement, crepitus, reduced range of motion, and tenderness. Radiographic features include joint space narrowing, osteophyte formation, subchondral sclerosis, and cysts. Osteoarthritis is the most common joint disease worldwide, leading to significant disability and impaired quality of life, especially in older adults. The knee and hip are the most frequently affected joints, but the hands, spine, and other joints can also be involved. The disease burden is substantial, with increasing prevalence due to aging populations and rising obesity rates. There is currently no cure, and management focuses on symptom relief and functional improvement through lifestyle modification, physical therapy, pharmacologic agents, and, in advanced cases, joint replacement.
Response 1 - What is PsA?	Psoriatic arthritis (PsA) is a chronic autoimmune inflammatory arthritis that occurs in some people who have psoriasis, a skin condition characterized by red, scaly patches. It is part of the family of spondyloarthropathies, which are inflammatory joint diseases associated with certain genetic factors. PsA can affect joints, tendons, and ligaments, and it may also involve the spine. Causes and risk factors: autoimmune origin - the immune system mistakenly attacks healthy joint tissue, causing inflammation. Genetic predisposition - certain genes, especially HLA-B27, increase risk. Psoriasis history - about 20-30% of people with psoriasis develop PsA. Environmental triggers - infections or trauma may trigger disease in genetically predisposed individuals. Symptoms - PsA symptoms can vary widely. Joint pain and stiffness - often affects fingers, toes, and larger joints asymmetrically. Swelling (dactylitis) - fingers or toes may become “sausage-like.” Nail changes - pitting, ridges, or separation of the nail from the nail bed. Skin symptoms - red, scaly patches typical of psoriasis. Enthesitis - inflammation at tendon or ligament attachment sites (e.g., Achilles tendon, plantar fascia). Fatigue and reduced function - chronic inflammation can lead to tiredness and mobility issues. Patterns of joint involvement: asymmetric oligoarthritis - few joints on one side of the body. Symmetric polyarthritis - similar to rheumatoid arthritis but less severe. Distal interphalangeal (DIP) joint arthritis - common in fingers and toes. Spondylitis - spine involvement causing stiffness and back pain. Arthritis mutilans - rare severe form causing joint destruction. Diagnosis: clinical assessment - joint and skin examination. Laboratory tests - no single test is definitive; may include markers of inflammation (ESR, CRP) and ruling out rheumatoid factor. Imaging - X-rays, MRI, or ultrasound can detect joint damage and inflammation. Treatment - the goal is to reduce inflammation, prevent joint damage, and improve quality of life. NSAIDs - relieve pain and stiffness. disease-modifying anti-rheumatic drugs (DMARDs) - methotrexate, sulfasalazine, to slow disease progression. Biologic therapies - TNF inhibitors, IL-17 or IL-23 inhibitors for moderate-to-severe cases. Physical therapy - maintains mobility and function. Lifestyle measures - weight management, exercise, and skin care. Prognosis: variable: some patients have mild disease; others may experience progressive joint damage. Early diagnosis and treatment are key to preventing disability. Can impact quality of life due to pain, fatigue, and skin lesions.
Response 2 - What is PsA?	Psoriatic arthritis (PsA) is a chronic inflammatory autoimmune disease that affects some people who have psoriasis, a condition characterized by red, scaly patches on the skin. While most people develop psoriasis first and then psoriatic arthritis years later, for some, the joint problems can appear before the skin issues. In PsA, the body's immune system mistakenly attacks healthy cells and tissues, leading to inflammation in the joints and an overproduction of skin cells. The disease can range from mild to severe, and without treatment, it can lead to permanent joint damage and disability. Symptoms of Psoriatic Arthritis: The symptoms of PsA can vary widely and often come and go in periods called flares. They can affect any part of the body, and often resemble those of rheumatoid arthritis, but with some key differences. Common symptoms include joint pain, stiffness, and swelling. This can affect joints on one or both sides of the body, including fingers, toes, knees, ankles, and the spine. Dactylitis: a painful, sausage-like swelling of an entire finger or toe. Enthesitis: pain and tenderness where tendons and ligaments attach to bones, most commonly in the heel (Achilles tendinitis) or the sole of the foot (plantar fasciitis). Nail changes: fingernails and toenails may develop tiny dents (pits), crumble, or separate from the nail bed. Fatigue: many people with PsA experience extreme tiredness. Eye inflammation: this can cause eye pain, redness, and blurry vision. Lower back pain: some people develop a condition called spondylitis, which causes inflammation of the joints in the spine. Psoriasis skin and nail symptoms: these typically accompany the joint symptoms. Causes and risk factors: the exact cause of psoriatic arthritis is not fully understood, but it is believed to be an autoimmune disease triggered by a combination of genetic and environmental factors. Risk factors for developing psoriatic arthritis include having psoriasis: this is the single greatest risk factor. Family history: many people with PsA have a parent or sibling with either psoriasis or psoriatic arthritis. Age: PsA most often occurs in adults between the ages of 30 and 55 years. Treatment: there is no cure for psoriatic arthritis, but treatments are available to manage symptoms and prevent joint damage. Treatment plans are often tailored to the individual and may include medications: This can include non-steroidal anti-inflammatory drugs (NSAIDs) for pain and inflammation, and more potent medications like disease-modifying antirheumatic drugs (DMARDs) and biologics to slow disease progression. Physical therapy and exercise: regular, low-impact exercise can help keep joints strong and improve overall health. Lifestyle modifications: managing stress, maintaining a healthy weight, and avoiding triggers like smoking can help reduce the frequency and severity of flares.
Response 3 - What is PsA?	Psoriatic arthritis is a chronic, immune-mediated inflammatory arthritis that occurs in individuals with psoriasis, affecting approximately 6-30% of patients with psoriasis, depending on the population studied. It is characterized by a heterogeneous clinical presentation, including peripheral arthritis (which may be asymmetric oligoarthritis or symmetric polyarthritis), axial involvement (spondylitis and sacroiliitis), enthesitis (inflammation at tendon or ligament insertions), dactylitis "sausage digits" and skin and nail changes such as pitting and onycholysis. The majority of patients develop psoriasis prior to the onset of joint symptoms, but in some cases, arthritis may precede or occur simultaneously with skin disease. Psoriatic arthritis is classified as a seronegative spondyloarthritis, typically lacking rheumatoid factor and anti-cyclic citrullinated peptide antibodies. Five clinical subtypes are recognized: distal interphalangeal predominant, asymmetric oligoarticular, symmetric polyarthritis, spondylitis, and arthritis mutilans. Comorbidities are common and include cardiovascular disease, diabetes, metabolic syndrome, uveitis, and inflammatory bowel disease. The pathogenesis involves genetic susceptibility (notably HLA alleles), environmental triggers, and dysregulation of innate and adaptive immune pathways, particularly involving TNFα, IL-17, and IL-23. Early recognition and treatment are critical to prevent irreversible joint damage and improve long-term outcomes. The American College of Rheumatology and the National Psoriasis Foundation recommend a combination of non-pharmacologic and pharmacologic therapies, including NSAIDs, conventional synthetic DMARDs, and biologic agents targeting TNFα, IL-17, and IL-23 pathways.

**Table 6:** Expert best/worst rankings of AI-generated rheumatology responses. After reviewing and rating the individual AI-generated responses for each question, reviewers were asked to identify the best and worst response for each disease-specific prompt “What is__?”. For each question, reviewers could select response 1, response 2, or response 3.

Question	Reviewer selection
Select the best response to “What is RA?”	Response 1, response 2, or response 3
Select the worst response to “What is RA?”	Response 1, response 2, or response 3
Select the best response to “What is OA?”	Response 1, response 2, or response 3
Select the worst response to “What is OA?”	Response 1, response 2, or response 3
Select the best response to “What is PsA?”	Response 1, response 2, or response 3
Select the worst response to “What is PsA?”	Response 1, response 2, or response 3
